# Fabrication and centeracterization of ordered CuIn_(1−*x*)_Ga_*x*_Se_2_ nanopore films via template-based electrodeposition

**DOI:** 10.1186/1556-276X-7-675

**Published:** 2012-12-17

**Authors:** Ming Li, Maojun Zheng, Tao Zhou, Changli Li, Li Ma, Wenzhong Shen

**Affiliations:** 1 Laboratory of Condensed Matter Spectroscopy and Opto-Electronic Physics and Key Laboratory of Artificial Structures and Quantum Control (Ministry of Education), Department of Physics, Shanghai Jiao Tong University, Shanghai, 200240, People’s Republic of China; 2 School of Chemistry & Chemical Technology, Shanghai Jiao Tong University, Shanghai, 200240, People’s Republic of China

**Keywords:** CuIn_(1−*x*)_Ga_*x*_Se_2_, nanopore films, electrodeposition, anodic aluminumoxide, annealing, 82.45.Yz, 81.05.Rm, 81.15.Pq, 81.40.Ef

## Abstract

Ordered CuIn_(1−*x*)_Ga_*x*_Se_2_ (CIGS) nanopore films were prepared by one-step electrodeposition based on porous anodized aluminum oxide templates. The as-grown film shows a highly ordered morphology that reproduces the surface pattern of the substrate. Raman spectroscopy and X-ray diffraction pattern show that CIGS nanopore films had ideal chalcopyrite crystallization. Energy dispersive spectroscopy reveals the Cu-Se phases firstly formed in initial stage of growth. Then, indium and gallium were incorporated in the nanopore films in succession. Cu-Se phase is most likely to act as a growth promoter in the growth progress of CIGS nanopore films. Due to the high surface area and porous structure, this kind of CIGS films could have potential application in light-trapping CIGS solar cells and photoelectrochemical water splitting.

## Background

In recent years, solar cells attract people’s attention for its clean and renewable properties [1]. Chalcopyrite CuInSe_2_/CuIn_(1−*x*)_Ga_*x*_Se_2_ (CIS/CIGS) thin films are considered as a promising candidate for solar cells since they have a high light absorption coefficient (about 10^5^ cm^−1^), good radiation, and thermal stability [2-7]. Also, CIGS has a direct and tunable bandgap range from 1.04 to 1.72 eV owing to the components of indium and gallium. Moreover, photoelectrochemical water splitting property of CIGS has been discussed in works in recent years [8,9]. Several methods have been reported to fabricate CIGS thin films such as co-evaporation, electrode position, selenization of sequentially stacked precursors, etc. [3,10-12]. A high conversion efficiency of 19.9% at laboratory scale was reported via a three-stage co-evaporation with a modified surface termination [3]. Also, the new record has been reported to achieving 20.3% last year [13]. Both of them have high conversion efficiency, but they all have the same disadvantages that the method is sophisticated and needs an expensive vacuum technology. However, electrodeposition is a competitive method that is economic and convenient. It also has high deposition speed and can prepare large area films [14]. Though the conversion efficiency of one-step electrodeposition is much lower than that of co-evaporation method, it can be improved by annealing and selenization.

As is well known, nanostructures can mostly improve properties of materials at a certain aspect [15-20]. In recent years, much effort has been devoted to fabricating CIS/CIGS nanowires and nanotubes, trying to improve cell properties through changing their microstructures [21-24]. Herein, we firstly fabricated CIGS nanopore films using one-step electrodeposition method based on anodized aluminum oxide (AAO) templates. Due to the high specific surface area and the porous structure, the ordered CIGS nanopore films could be used in light-trapping solar cells and photoelectrochemical water splitting. AAO templates are used to confine the structure of the film during the process of growth. The film, after being annealed at 550°C, shows a better performance in crystallization through analyzing by Raman spectroscopy and X-ray diffraction. Mechanism of deposition has also been discussed.

## Methods

**Figure 1:**
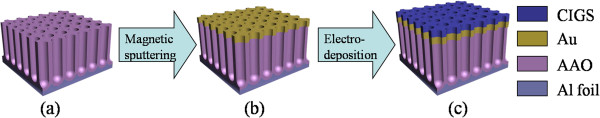
**Fabrication process of ordered CIGS nanopore films.** (**a**) AAO template with Al foil at the bottom and surrounding on its outside edge. (**b**) Au film sputtered on the top of the AAO template. (**c**) Ordered CIGS nanopore film deposited on the Au-coated substrate.

The fabrication process of CIGS nanopore film is shown schematically in Figure 1. AAO templates have been used as the substrate in the experiment, and the AAO templates were prepared by a two-step method which was described in our previous work [25]. Anodization of Al foil was carried out in 0.25 M H_3_PO_4_ electrolyte (C_2_H_5_OH/H_2_O = 1:4 *v*/*v*) at 195 V while the temperature was kept at −5°C. Then, the as-prepared AAO films were immersed in 5 wt.% phosphoric acid at 45°C for about 40 min to get a proper pore diameter. A layer of gold was sputtered on the AAO template with the power of 100 W for 3 min. CIGS thin films were deposited on Au-coated AAO template via a three-electrode configuration. It consists of a reference electrode (saturated calomel electrode (SCE)), a counter electrode (graphite), and the working electrode (Au-coated substrate). The electrodepositing bath contains 2 mM CuCl_2_, 6 mM InCl_3_, 16 mM GaCl_3_, 4 mM H_2_SeO_3_, and 0.17 M LiCl. LiCl serves as the supporting electrolyte. The pH was adjusted to 2.2 by NaOH buffer. The experiment with the applied potential of −0.8 V (vs. SCE) was carried out for 20 min at room temperature. The as-prepared CIGS films were rinsed with deionized water and dried with nitrogen. Then, the films were annealed at different temperatures with the heating rate of 10°C/min in a vacuum tube furnace for 30 min. 

The morphology of as-prepared and annealed CIGS films was observed by field emission scanning electron microscopy (FE-SEM; Philips Sirion 200, Philips, Netherlands). The composition was investigated by energy-dispersive X-ray spectrometer (EDS) system attached to FE-SEM. The Raman spectra were measured by LabRam HR 800 UV system (Jobin Yvon, France). The crystallographic structure was determined by X-ray diffraction (XRD; D8 DISCOVER X-ray diffractometer, Bruker, Germany) with Cu Kα radiation (*λ* = 1.54*Å*).

## Results and discussion

### Surface morphology

**Figure 2:**
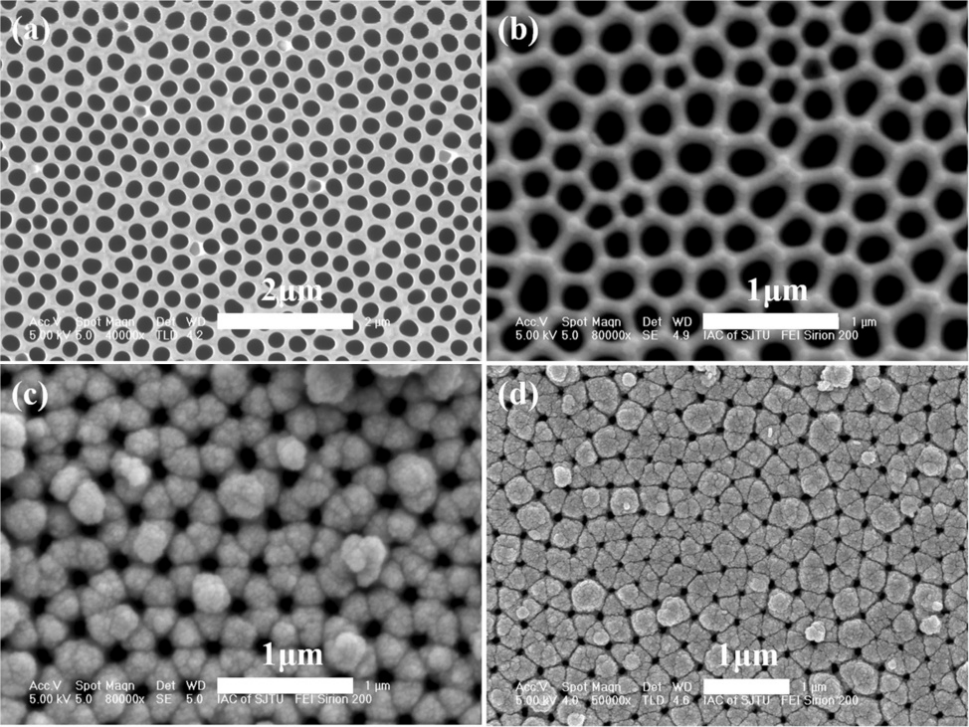
**FE-SEM images of AAO template, Au-coated AAO template, and as-grown and annealed CIGS nanopore films.** (**a**) Top view of the AAO template prepared by high-field anodization method. (**b**) Au-coated AAO template after broadening the pores. (**c**) As-grown CIGS nanopore film deposited with aqueous solution of 2 mM CuCl_2_, 6 mM InCl_3_, 16 mM GaCl_3_, and 4 mM H_2_SeO_3_. (**d**) CIGS nanopore film annealed at 550°C.

Figure 2a shows a FE-SEM image of typical AAO template prepared by a two-stepoxidization method. From the figure, we can see the typical hexagonally arranged shape of the pore of AAO template. The average diameter of nanopores is about 220 nm, which could be adjusted to about 250 nm by immersing in 5 wt.% phosphoric acid for 40 min. Au-coated AAO template with a diameter of 242 nm has been shown in Figure 2b, corresponding to the diameter of AAO template after being adjusted. Figure 2c is the FE-SEM image of as-grown CIGS nanopore film deposited on Au-coated AAO membrane. It can be seen in the figure that the as-grown CIGS nanopore film has the same morphology with AAO substrate, ordered and hexagonally arranged, and consists of small grains. Figure 2d shows the morphology of CIGS nanopore film annealed at 550°C. It is similar with the as-grown films in shape, but the surface is getting smoother and the grain size is much bigger. The pore diameters of as-grown and annealed films are 131 and 89 nm, respectively. Compared with thatof AAO substrate, they are much smaller, indicating a thickness limit of CIGS nanopore films.

**Figure 3:**
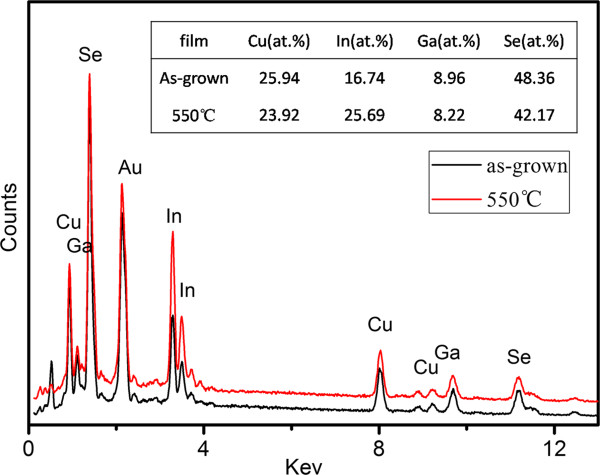
EDS spectrum of as-grown CIGS nanopore films and that annealed at 550°C.

Figure 3 displays the EDS spectrum of as-grown films together with that annealed at 550°C. From the spectrum, energy response of the four elements including copper, indium, gallium and selenium can be easily recognized. Through the table inserted in Figure 3, we know the as-grown film is approximately equal to the ideal stoichiometric ratio of chalcopyrite CIGS films with the Cu, In, Ga, and Se atomic ratio of 1:0.65:0.35:1.86. However, the ratio has been slightly changed after the film had been annealed. With the annealing process, the component of selenium has clearly decreased. Meanwhile, the component of indium has increased. The result may suggest producing a film with higher selenium content or adding a selenium source in the tube furnace to maintain the stoichiometric ratio.

### Structure centeracterization

**Figure 4:**
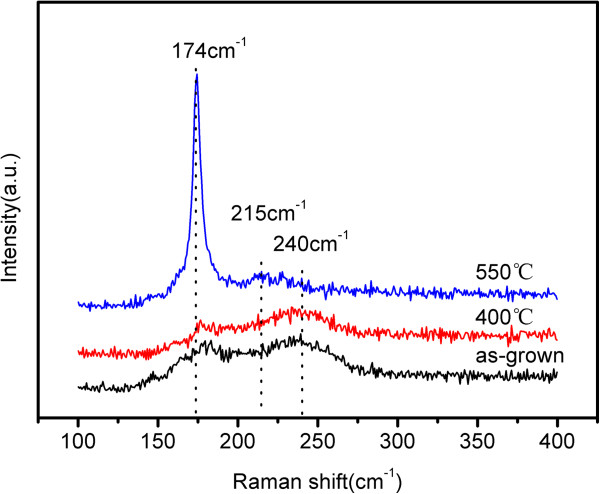
Evolution of Raman spectra of CIGS nanopore films.

**Figure 5:**
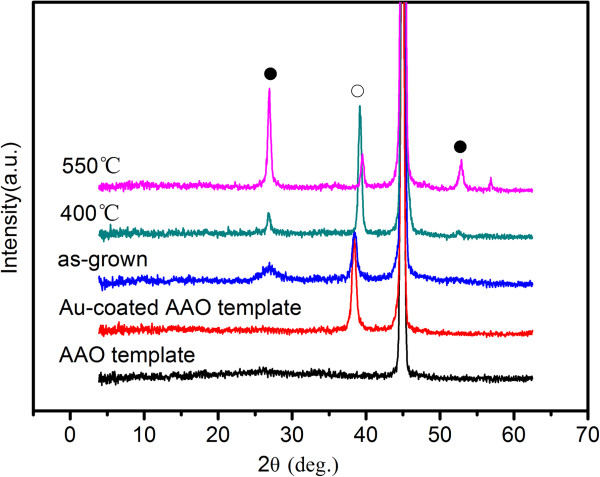
**XRD patterns of as-grown CIGS nanopore film and samples annealed at 400°C and 550°C, respectively.** White circle indicates Au; black circle, chalcopyrite Cu(In_0.7_Ga_0.3_)Se_2_.

Figure 4 shows the Raman spectra of CIGS nanopore films prepared at room temperature and annealed at 400°C and 550°C in the Stocks frequency range from 100 to 400 cm^−1^. For CuInSe_2_, CuGaSe_2_ and CIGS are all A^I^B^III^C_2_^VI^ chalcopyrite compounds; the active vibrations of these compounds should be very close to each other. The spectrum of as-grown films has two broad peaks at about 180 and 240 cm^−1^, in agreement with the A_1_ and E mode frequencies at 184 and 239 cm^−1^ of CuGaSe_2_ obtained by Rincon and Ramirez [26]. The spectrum of the films annealed at 400°C is similar to that of as-grown films. However, when the annealing temperature reached 550°C, the peak at 240 cm^−1^ disappears and replaced by a broad peak at about 215 cm^−1^, which is in agreement with the B_2_ mode in reports [26,27]. Since the peak at 240 cm^−1^ is indexed by elementary selenium due to trigonal selenium [27], the result indicate the decrease of selenium in the films annealed at 550°C, consistent with that of the EDS spectrum (Figure 3). It cannot be ignored that there is a strong peak at 174 cm^−1^ of films annealed at 550°C, corresponding to the A_1_ mode of chalcopyrite compounds. With the full width at half maximum (FWHM) value of 7 cm^−1^, it indicates that the films annealed at 550°C are pure chalcopyrite CIGS with improved crystallinity. XRD patterns of CIGS nanopore films were shown in Figure 5. Spectra of Au-coated and pure AAO templates were measured for comparison. The as-grown CIGS films show chalcopyrite CIGS structure as seen from the XRD spectrum because there is a broad peak at 26.92° in agreement with (112) reflection (PDF#35-1102). At the temperature of 400°C, the peak has become sharpened. When the temperature increased to 550°C, the peak at 26.92° has been more prominent, and a peak at 52.88° corresponding to (312) reflections of chalcopyrite CIGS structure has arisen. The most prominent peak at about 45° is indexed by the reflection of Al_2_O_3_, which conceals the (220) reflection of CIGS. From the XRD patterns, it shows that thermal treatment improves crystallinity of the films, and the size of grains increases after being annealed due to the decrease in FWHM of the diffraction peaks, consistent with the result of Raman spectra. 

### Process of growth

**Figure 6:**
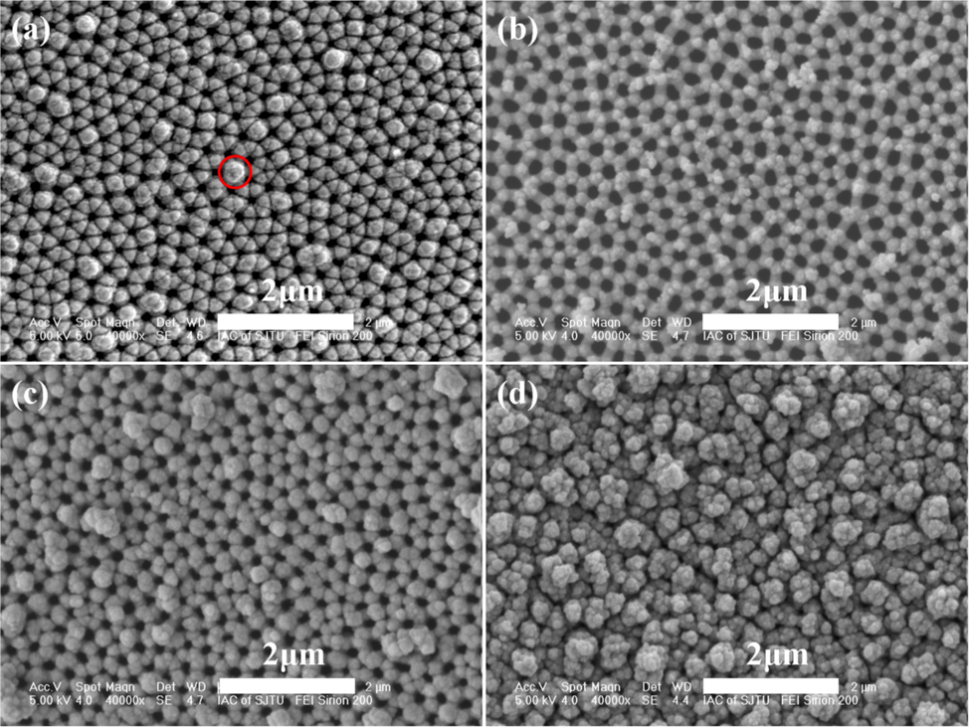
**FE-SEM images of CIGS nanopore films deposited under different times.** (**a**) 30 s, (**b**) 90 s, (**c**) 600 s, and (**d**) 1,200 s.

Figure 6 shows a set of FE-SEM images of CIGS nanopore films deposited for different times. From the SEM images and EDS spectra, the process of growth of CIGS nanopore films can be qualitatively discussed. In Figure 6a,b, grains are easily deposited on the corner of every single hexagonal Au-coated substrate for the first 30 to 90 s. When the deposition time continues to 10 min (see Figure 6c), the as-grown film gradually covers the surface of Au-coated substrate. When the films keep on depositing, grains will combine together to form clusters. Then, the clusters grow bigger, and finally, a thin film without porous structure was formed (see Figure 6d). In addition, the morphology of Figure 6d is different from that of Figure 2c because the pores of the substrate have not been broadened.

Table 1 displays the elementary component of Cu, In, Ga, and Se associated with the films shown in Figure 6. It is obvious that Cu and Se are firstly deposited when the three-electrode configuration works for 30 s. It should be noted that this EDS spectrum was investigated at such positions marked with a circle in Figure 6a. Most area only shows the energy reflection of Cu. Subsequently, In and Ga were incorporated in the film by 90 and 600 s, respectively. The component of In and Ga increases when the electrode keeps on working. The reduction succession corresponds to the standard reduction potential values of Se^4+^/Se, Cu^2+^/Cu, In^3+^/In, and Ga^3+^/Ga which are +0.740, +0.342, −0.338, and −0.523 V vs. standard hydrogen electrode, respectively. According to the report of Saji et al. [14], the electrochemical mechanism of CIGS deposition is not very different from that of CIS. The early stages of the CIS film growth were dominantly affected by Cu-rich phases. The deposition of Se did not occur separately but only when the binary Cu-Se phases have been deposited [28]. The formed Cu-Se phase provided active sites for the In incorporation [29]. Also, Calixto et al. reported that In incorporation occurs by reacting with H_2_Se formed by previous Cu-Se phase [30]. Another work reported by Lai et al. [31] suggests an underpotential deposition mechanism that In^3+^ and Ga^3+^ reduction occurs by surface-induced effect from Cu_3_Se_2_ and/or reaction with H_2_Se. Therefore, Cu-Se phases are necessary in the incorporation of In and Ga during the deposition of CIGS, which results in this reduction succession as shown in Table 1. 

**Table 1:** Elementary component of Cu, In, Ga, and Se in the as-grown CIGS nanopore films

**Deposition time(s)**	**Cu (at.%)**	**In (at.%)**	**Ga (at.%)**	**Se (at.% )**
30	44.44	-	-	55.56
90	29.29	17.54	-	53.17
600	26.80	14.39	5.02	53.79
1,200	24.24	23.79	6.51	45.46

## Conclusions

In summary, we firstly fabricated highly ordered CIGS nanopore films. The deposited film reproduced the morphology of the AAO substrate. With heat treatment, the CIGS nanopore films present an almost pure chalcopyrite nanocrystal. Moreover, Cu-Se phases firstly occur during growth of the film. Then, In and Ga incorporated in the films through reactions with Cu-Se phases. This large-scale ordered CIGS nanopore films could be used in light-trapping CIGS solar cells and photocatalytic hydrogen generation.

## Abbreviations

AAO: Anodized aluminum oxide; CIS/CIGS: CuInSe_2/_CuIn_(1−*x*)_Ga_*x*_Se_2_; EDS: Energy-dispersive X-ray spectrometer; FE-SEM: Field emission scanning electron microscopy; FWHM: Full width at half maximum; SCE: Saturated calomel electrode; XRD: X-ray diffraction.

## Competing interests

The authors declare that they have no competing interests.

## Authors’ contributions

ML participated in the design of the study, carried out the experiments, performed the statistical analysis, as well as drafted the manuscript. MJZ participated in the design of the study, provided the theoretical and experimental guidance, performed the statistical analysis, and revised the manuscript. TZ helped in the experiments and statistical analysis. LM participated in the design of experimental section and offered help in the experiments. WZS provided the experimental apparatus. All authors read and approved the final manuscript.
